# A Comparison of Partial Discharge Sensors for Natural Gas Insulated High Voltage Equipment

**DOI:** 10.3390/s20164443

**Published:** 2020-08-09

**Authors:** Phillip Widger, Daniel Carr, Meirion Hills, Alistair Reid

**Affiliations:** Advanced High Voltage Engineering Research Centre, School of Engineering, Cardiff University, Cardiff CF24 3AA, UK; widgerp@cardiff.ac.uk (P.W.); HillsMT@cardiff.ac.uk (M.H.); ReidA3@cardiff.ac.uk (A.R.)

**Keywords:** gas insulated transmission lines (GIL), partial discharge (PD), sulphur hexafluoride (SF_6_), carbon dioxide (CO_2_), technical air, ultra high frequency (UHF) sensors, high frequency current transformer (HFCT)

## Abstract

The research in this paper consists of practical experimentation on a gas insulated section of high voltage equipment filled with carbon dioxide and technical air as a direct replacement to sulphur hexafluoride (SF_6_) and analyses the results of PD measurement by way of internal UHF sensors and external HFCTs. The results contribute to ongoing efforts to replace the global warming gas SF_6_ with an alternative such as pure carbon dioxide or technical air and are applicable to mixtures of electronegative gases that have a high content of buffer gas including carbon dioxide. The experiments undertaken involved filling a full-scale gas insulated line demonstrator with different pressures of CO_2_ or technical air and applying voltages up to 242 kV in both clean conditions and particle contaminated conditions. The results show that carbon dioxide and technical air can insulate a gas section normally insulated with SF_6_ at phase-to-earth voltage of 242 kV and that both HFCT and UHF sensors can be used to detect partial discharge with natural gases. The internal UHF sensors show the most accurate PD location results but external HFCTs offer a good compromise and very similar location accuracy.

## 1. Introduction

In the high voltage power industry sulphur hexafluoride (SF_6_) is currently used to insulate high voltage equipment worldwide such as gas insulated transmission lines (GIL) and gas insulated switchgear (GIS). SF_6_ is a man-made gas and has a global warming potential 23,500 times [1] that of carbon dioxide (CO_2_) when released into the atmosphere, along with an estimated atmospheric lifetime of 3200 years [2] making it one of the most potent greenhouse gases known to humankind. The use of SF_6_ is increasing in the UK [3] and hence is highly regulated. The use of SF_6_ is restricted to industries where no alternatives exist, such as the power industry. Considering this, high voltage equipment manufacturers and research institutions worldwide have been trying to find a suitable alternative to replace SF_6_ in all current equipment required for use in the energy sector. Present solutions are focused on the use of natural gases such as carbon dioxide, nitrogen or technical air at higher pressures to match the insulation and interruption capability of SF_6_ or the use of electronegative gas mixtures with high mixture ratios of the same natural buffer gases [4,5,6,7,8,9].

In order to directly replace SF_6_ with an alternative natural gas or electronegative gas mixture it is important to understand how these gases perform electrically in GIS and GIL equipment prior to a breakdown event where an electrical arc crosses the insulation gas from inner conductor at high voltage to outer enclosure which is at ground potential. To detect and compare the electrical performance of the insulation gas or equipment prior to breakdown, partial discharge (PD) sensors can be used to detect very small electrical discharges which are formed around areas of insulation degradation or high electric field stresses at lower voltages. In gas insulated equipment it is important to determine the level of PD as it can help identify insulation performance weakness or areas of high electrical field stress where sections, solid insulation components and gas insulation may need to be replaced or the installation rectified before an electrical breakdown causes equipment downtime and perhaps costly unplanned network outages.

Two PD sensors can be used to locate the cause of PD within a GIL by calculating the time difference of arrival of a PD signal and hence simplifying the RF wave propagation to a 1-dimensional problem. Some common time-of-flight methods of PD detection in SF_6_ include UHF sensors and acoustic sensors. In this paper, internal UHF sensors are compared with external HFCTs. An external capacitively coupled PD measurement through a dividers HV capacitors or power separation filter is also part of the high voltage test transformer arrangement. These sensors are primarily used in this paper to detect PD in a gas insulated section filled with CO_2_ or technical air and show whether traditional sensors, primarily associated with PD detection in SF_6_-insulated equipment, can be used accurately to detect and locate PD in alternative gases. For practical implementation onto the network PD detection must consider sensor design including size and shape, signal delay through solid barriers or angles and detection of the signal and recognising patterns. In this paper, a straight section of gas insulated line is used to study the PD time-of-flight measurements but it is important to note that previous studies have conducted research regarding the effects of signal delay through solid dielectric barriers [10], effects of 90° bends and L-shapes in GIS [11] and the specific shape and sizes of sensor design for PD applications [12]. Novel sensor designs for PD currently in development include antennas using magnetic field detection [13] and HFCTs [14], which are not currently used on the network. Additional constraints to be considered for PD detection include optical placement of sensors [15] and signal processing/pattern recognition of partial discharge signals [16].

## 2. Practical Test Arrangement and PD Sensors

**Figure 1:**
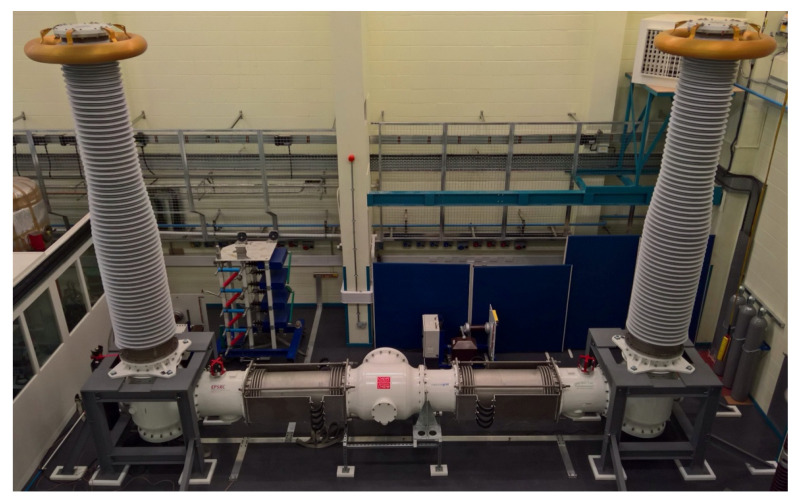
Gas Insulated line demonstrator constructed and installed at Cardiff University.

**Figure 2:**
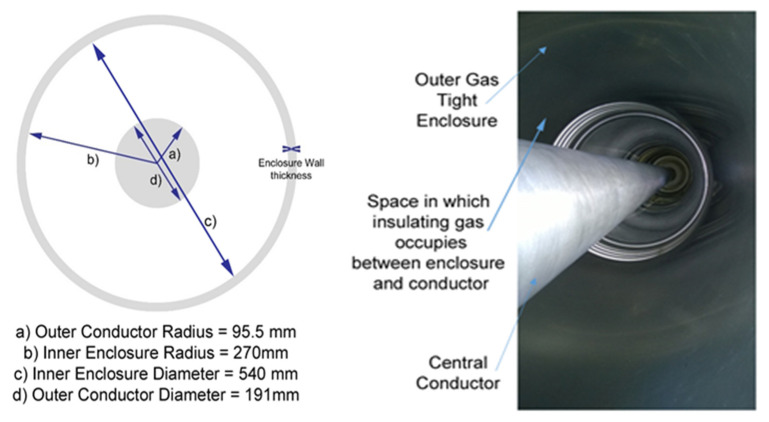
Gas insulated line conductor and enclosure dimensions.

During the practical experimentation undertaken the following test arrangement was utilised to obtain the partial discharge measurements shown in the next section. The full-scale gas insulated sections, shown in Figure 1, were arranged and constructed into a 420 kV, 7 m length of gas insulated line with a high voltage air/gas insulated bushing termination at either end. The central section was a separate gas zone from both bushings but was electrically connected to the rest of the demonstrator via the central conductor at the same high voltage potential as the bushing conductors and terminations. The outer enclosure of the GIL demonstrator was electrically continuous throughout and was connected to ground. The enclosure and conductor dimensions are shown in Figure 2. The central gas zone, which was the primary gas zone tested, consisted of a 4.915 m conductor suspended between two epoxy resin cone spacers (which seal the gas zone at either end) and an outer enclosure length of 5.275 m.

The gas insulated line demonstrator had a rated voltage of 420 kV with nominal system phase to earth voltage and normal partial discharge test voltage level, as shown in Table 1 considering the IEC/BS EN standard 62271-204 [17] and 62771-203 [18]. Partial discharge measurements taken using commercially available systems for the GIL demonstrator filled with CO_2_ at varying voltage levels can be found in reference [19] to experimentally show the insulation strength of alternative gases and the capabilities of traditional sensors with AC cycle analysis. The following article shows novel PD measurements using non-commercial setups, sensors and analysis to determine fault locations and analyze an author-developed location script with results which do not form part of reference [19].

**Figure 3:**
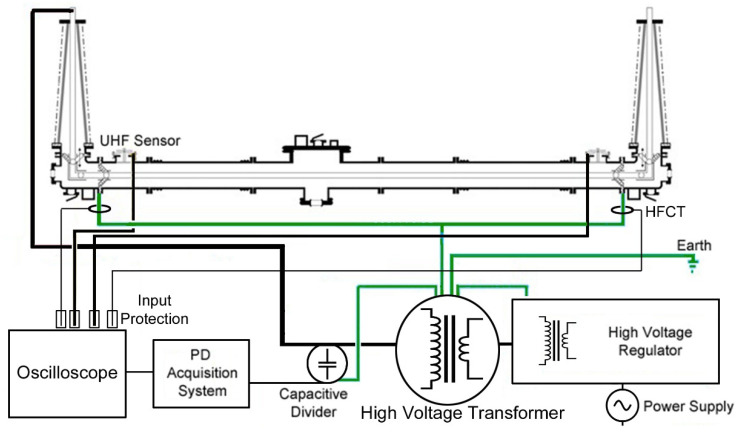
Gas insulated line high voltage connection and capacitively coupled PD measurement through a High Voltage divider’s capacitors test arrangement.

**Figure 4:**
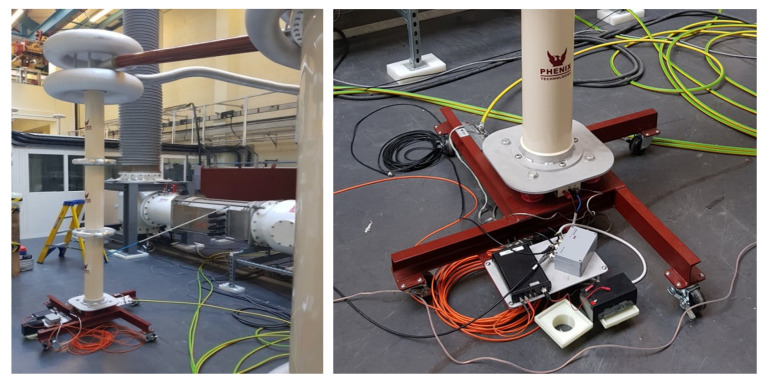
Optically isolated PD measurement system coupled to a 300 kV AC HV capacitive divider.

The GIL demonstrator had a voltage applied to the inner conductor using the test arrangement shown in Figure 3 through a high voltage test transformer with a maximum AC output of 300 kV and a low partial discharge output. The output of this transformer was limited by an inductance and connected to a coupling capacitive divider which allows for direct partial discharge measurements to be obtained for the whole system via a commercially available optically isolated PD system shown in Figure 4. The output of this capacitively coupled PD signal through a divider’s HV capacitors can be recorded by directly connecting them to a high bandwidth oscilloscope.

**Figure 5:**
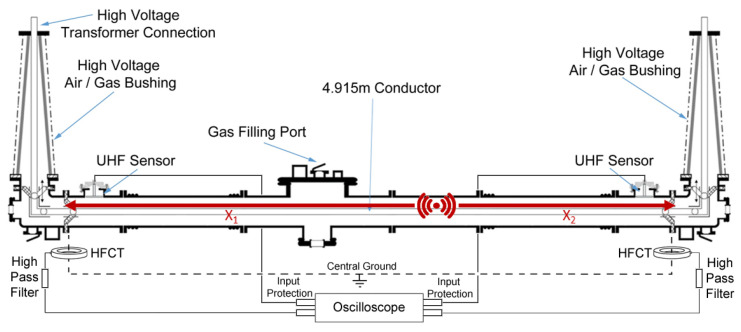
Gas insulated line partial discharge sensor arrangement, data acquisition and measurement.

**Figure 6:**
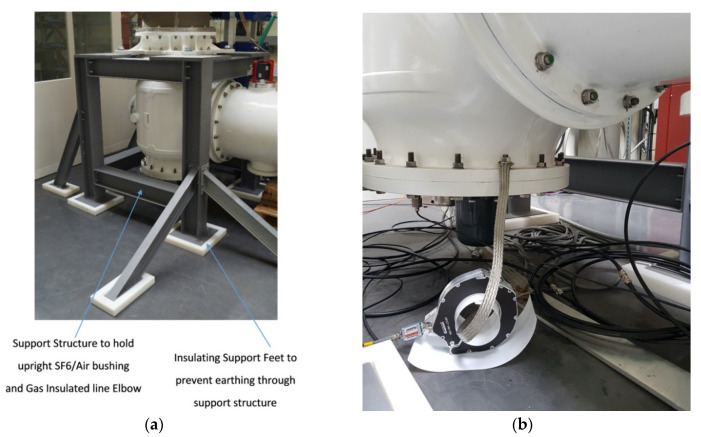
(**a**) Metal Support Structure with insulated pad mounted feet; (**b**) One of two split core HFCT partial discharge sensors installed on a single earth strap connection.

Partial discharge measurements were also undertaken using external high frequency current transformers (HFCTs) and internal UHF capacitive plate sensors. Two split core HFCTs were installed on the only two earth straps for the gas insulated line demonstrator at either end of the central section as shown in Figure 5 and Figure 6b. The whole of the gas insulated line demonstrator was installed on a metal support structure as would normally be the case in substations, however, the support structure was insulated from the floor using solid pad-mounted insulation feet, shown in Figure 6a. This insulated support structure was not bolted to the floor therefore ensuring the only ground of the outer enclosure was provided via the central star grounding point of the transformer as shown in Figure 3. This dedicated ground ensured that the HFCTs were installed on the only two points of grounding for the whole GIL demonstrator, thereby minimising noise and avoiding multiple grounding loops as are common in substations but could be improved for the future using this method of insulation. The HFCT sensors have a bandwidth of 100 kHz to 50 MHz and a sensitivity of 3.1 Ω [20] and were both connected to identical 100 kHz high pass filters [20]. The output of the HFCT filters were then connected to identical 20 m lengths of double screened BNC cable to a digital oscilloscope which has a bandwidth of 1 GHz and additional voltage limiting protection on the input of each channel.

**Figure 7:**
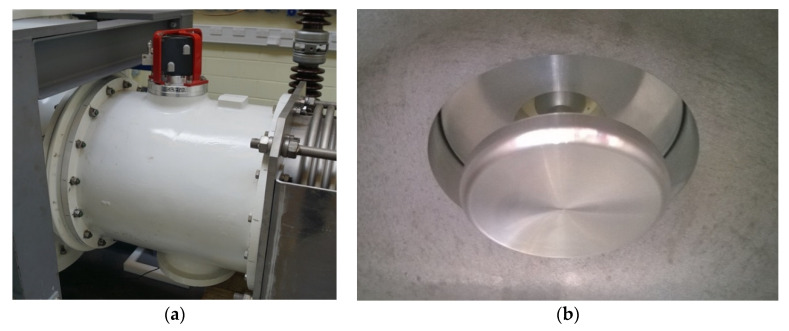
(**a**) UHF plate coupling sensor on gas insulated line enclosure section; (**b**) One of two GIL internal UHF plate coupling sensors.

Internal UHF capacitive plate sensors were also installed at either end of the inside of the gas insulated line demonstrator as shown in Figure 5. The outside of a UHF sensor gas section is shown in Figure 7a, and an internal plate sensor is shown in Figure 7b. The UHF sensors have a frequency range of 200 to 1500 MHz [21] and are both connected via identical RG213 cables and N-type connectors of 15.2 m [22] to a digital oscilloscope which has a bandwidth of 1 GHz. Additional UHF attenuators are also added to the input of each channel, reducing the amplitude of large UHF signals to within the limits of the oscilloscope input and providing some protection to the oscilloscope channels by providing a resistive path to ground in the event of a flashover or electrostatic discharge.

It is also worth noting that the results in the next section were all taken using the described GIL demonstrator transmission line which does not have a load at one open terminal end. It is also noted that there will be a difference in time and voltage for HFCT and UHF sensor measurements for each PD event which is introduced due to sensitivity, input protection components on the oscilloscope, different lengths of connected cables and additional high pass filters on the outputs of the high frequency current transformers.

The gas insulated sections utilised in this study were actual GIS sections donated by the National Grid of the same design and construction as those normally filled with SF_6_ gas and are currently in operation on the UK power network. In this paper, the practical gas insulated line demonstrator was filled with either pure nitrogen, pure carbon dioxide or technical air as a direct replacement for SF_6_. The technical specification of the CO_2_ used was 99.8% purity [23], the nitrogen purity used was 99.998% (including argon) [24] and the purity of the technical air used was 21% ± 0.5% oxygen and the remainder nitrogen [25] to a pressure of 3, 4 and 5 bar. The normal operating pressure of this gas insulated equipment has a minimum filling pressure of 2.9 bar when filled with SF_6_ at 20 °C. The gas sections are evacuated using a vacuum pump to 0.1 mbar prior to each gas filling and the central section and each of the two bushing sections are filled using the same identical gas to the same pressure. The gas insulated demonstrator was tested as both a clean system and as a particle-contaminated system with 10 × 1 mm diameter stainless steel spheres placed within the central gas insulated enclosure [26].

## 3. Results

### 3.1. Partial Discharge Measurements

The first stage of practical testing involved raising the voltage potential of the central conductor in the uncontaminated gas insulated demonstrator, to re-create normal operating conditions, when filled with different natural gases at different pressures. In later stages of the work, the central section of the gas insulated line was contaminated with 10 × 1 mm diameter stainless steel spheres placed as free particles randomly within the central gas insulated enclosure, which changes at the start of each test according to their random dispersion due to the applied voltage [26]. As the voltage was raised, the measurements taken on the oscilloscope were triggered as partial discharge occurred. In some gases at higher gas pressures, there are very few PD events, so it is not possible to record results but for these eventualities the pressure of the gas was lowered to a point where a statistically significant volume of PD data might be recorded. The practical experimentation undertaken showed the withstand voltages for the gas insulated line for various gases at different pressures shown in Table 2. It was found that the weakest insulation gas out of the three was pure nitrogen, which was the only gas to break down between the conductor and enclosure at 211 kV. Pure carbon dioxide and technical air were tested at 5, 4 and 3 bar, and were found to withstand 242 kV, which is the normal phase to earth system voltage level of this gas insulated equipment when insulated with SF_6_ at 2.9 bar. This means that there was a considerable safety margin built into this equipment to ensure it does not fail during the operation on the network, as it is known that SF_6_ has an insulation strength approximately three times that of normal air (not technical air) [8]. The insulation strength and therefore the different PD results of each alternative natural gas are due to the individual critical reduced electric field strength of each gas [8] and the method in which streamers and leader channels form and how ‘brittle’ the gas is to allow streamer development [27].

**Figure 8:**
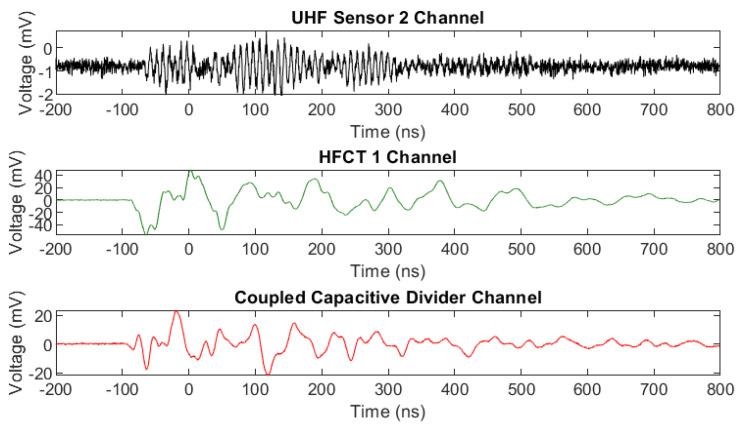
Simultaneously measured UHF, HFCT and HV coupled capacitive divider PD signals for a CO_2_ filled system at 3 bar with particle contamination and an applied voltage of 85 kV.

In Figure 8, measured signals from one single PD event are shown for the GIL demonstrator when it was filled with 3 bar CO_2_ with contaminated particles. The recorded results show that all of the sensors including the internal UHF sensors similar to those in [28,29], the external HFCTs and the capacitively coupled PD measurement through a divider’s HV capacitors all confirm the same event and their own respective outputs are shown depending on their frequency range and sensor output in voltage. Many similar events were recorded for both carbon dioxide and technical air; however, it was not possible to obtain PD measurements for pure nitrogen due to the proximity of PD inception to the breakdown voltage which occurred across the clean gas gap, making it very difficult to obtain multiple stable PD event measurements.

**Figure 9:**
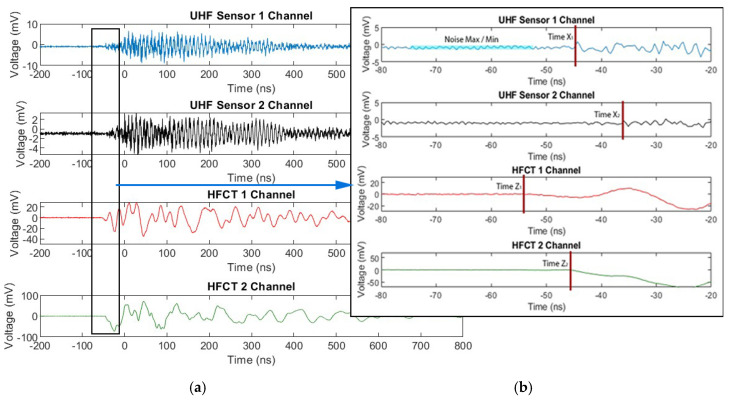
Recorded UHF and HFCT PD signals for a CO_2_ contaminated system at a pressure of 3 bar, applied voltage 85 kV (**a**) Full waveform; (**b**) Voltage rise point of each sensor.

In Figure 9a, an example is shown of the measurements recorded using the oscilloscope when a single PD event is recorded from both of the UHF sensors and both of the HFCTs at either end of the gas insulated line demonstrator. From the measurements undertaken it is possible to quantify the arrival time of each signal from each one of these four sensors. In order to find the start time of the signal it is first required to take a sample of the beginning of the measured trace in order to give the maximum and minimum noise of each channel which means that the error for each signal and solution found is different depending on the level of noise. Using these voltage noise values it is possible to determine the first voltage instance of any value above or below this maximum and minimum noise threshold which can then be used to determine the time at which the start of the sensor picks up a PD event as shown in Figure 9b. Using both UHF sensors and both HFCTs, the start time of each channel can be used to calculate the time difference at which the signal is detected by each PD detection method, therefore, allowing for a location between the two sensors to be determined. If equation 1 is used to determine the time-of-flight then a time difference between the arrival of the signal for each detection method can be estimated for each PD event.

Time Difference between Signals = X_1_ − X_2_ (1)

X_1_ is the time at which the signal arrives at the UHF or HFCT sensor on the left of the GIL, X_2_ is the time at which the signal arrives at the UHF or HFCT on the right of the GIL. If the outcome of equation 1 is negative then the PD event occurred on the left side of the GIL, if the equation gives a positive number then the PD event is determined to have originated on the right side of the GIL, if the outcome is equal to zero then the PD event originated in the midpoint of the GIL. It is assumed that the signals are recorded simultaneously and that all signals are from the same source/event.

### 3.2. Results of Time of Flight Calculations

**Figure 10:**
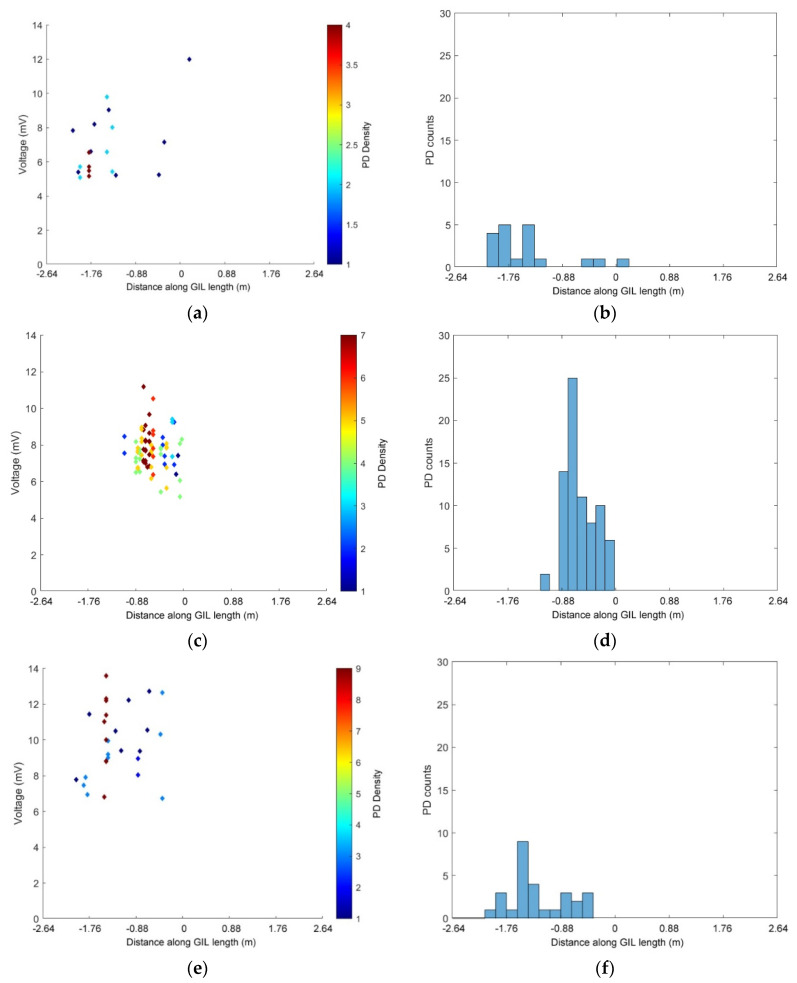
PD UHF sensors in technical air for a clean system: (**a**,**b**) applied voltage 210 kV at 3 bar; (**c**,**d**) applied voltage 200 kV at 5 bar; (**e**,**f**) applied voltage 231 kV at 5 bar.

**Figure 11:**
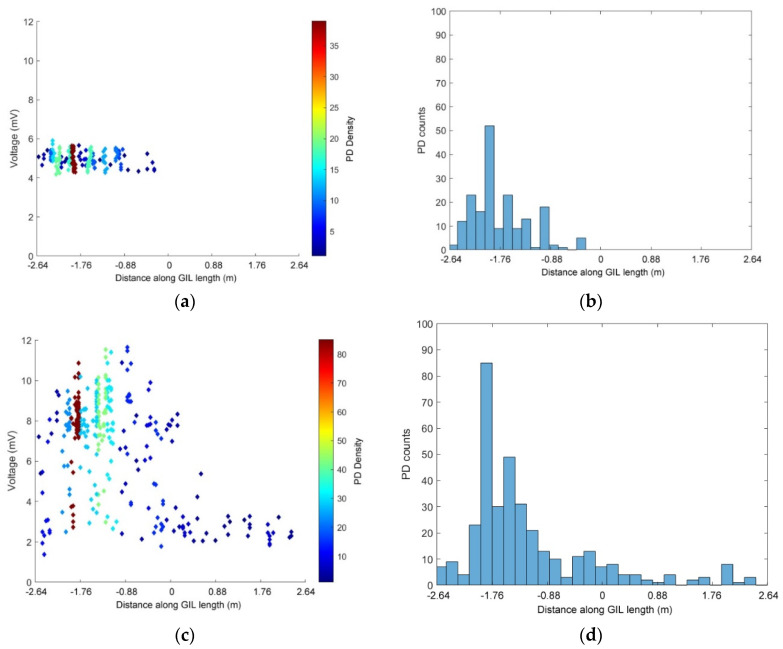
PD UHF sensors in CO_2_ at 3 bar, (**a**,**b**) 150 kV applied voltage in a clean system; (**c**,**d**) 85 kV applied voltage in a contaminated system.

**Figure 12:**
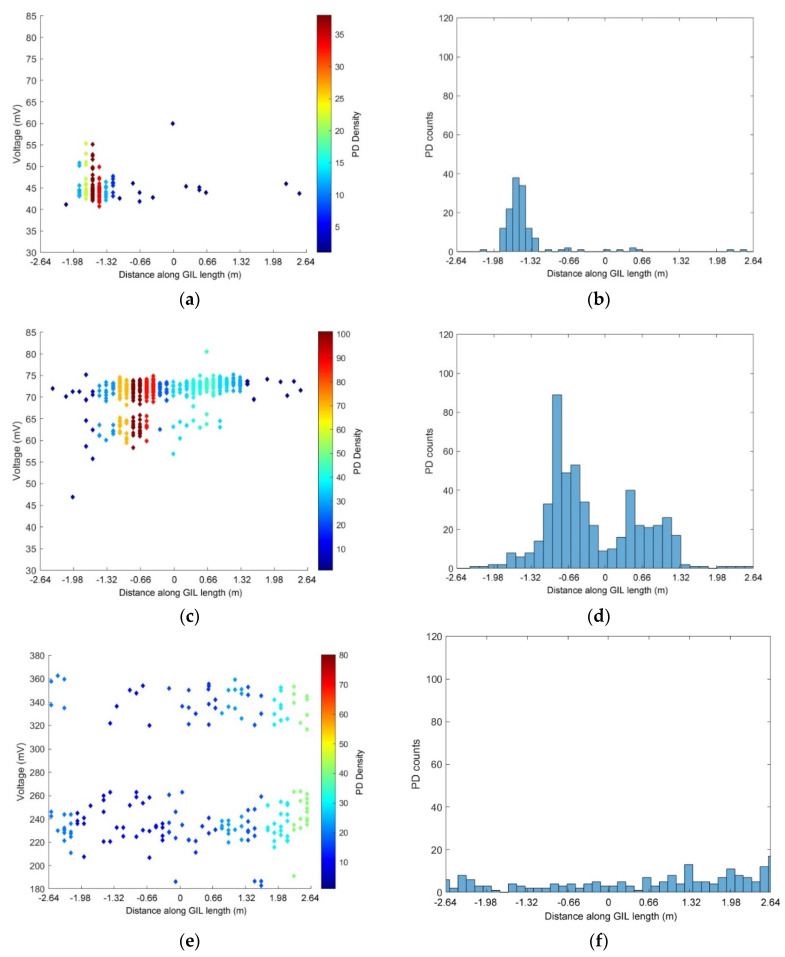
PD HFCT sensors in CO_2_ at 3 bar with a particle contaminated system, (**a**,**b**) 75 kV applied voltage; (**c**,**d**) 85 kV applied voltage; (**e**,**f**) 100 kV applied voltage.

**Figure 13:**
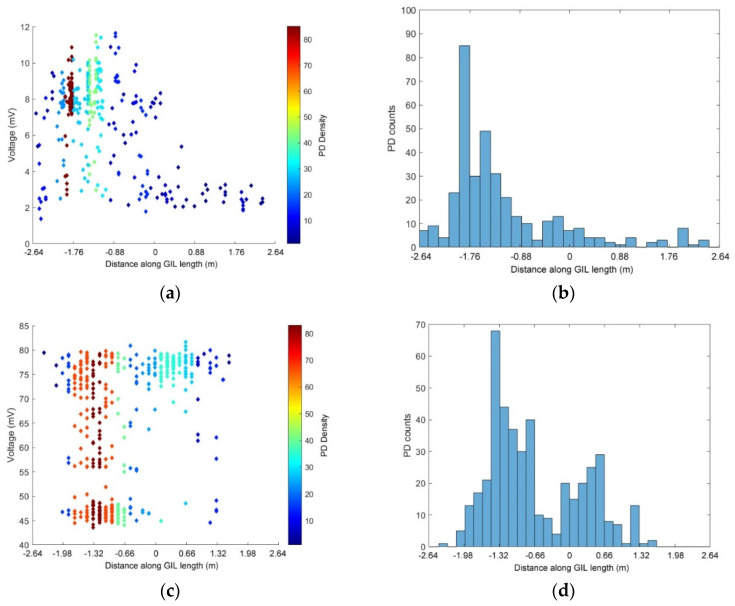
(**a**,**b**) PD UHF sensors in CO_2_ at 3 bar, 85 kV applied voltage with a particle contaminated system; (**c**,**d**) PD HFCT sensors in CO_2_ at 3 bar, 85 kV applied voltage with a particle contaminated system.

**Figure 14:**
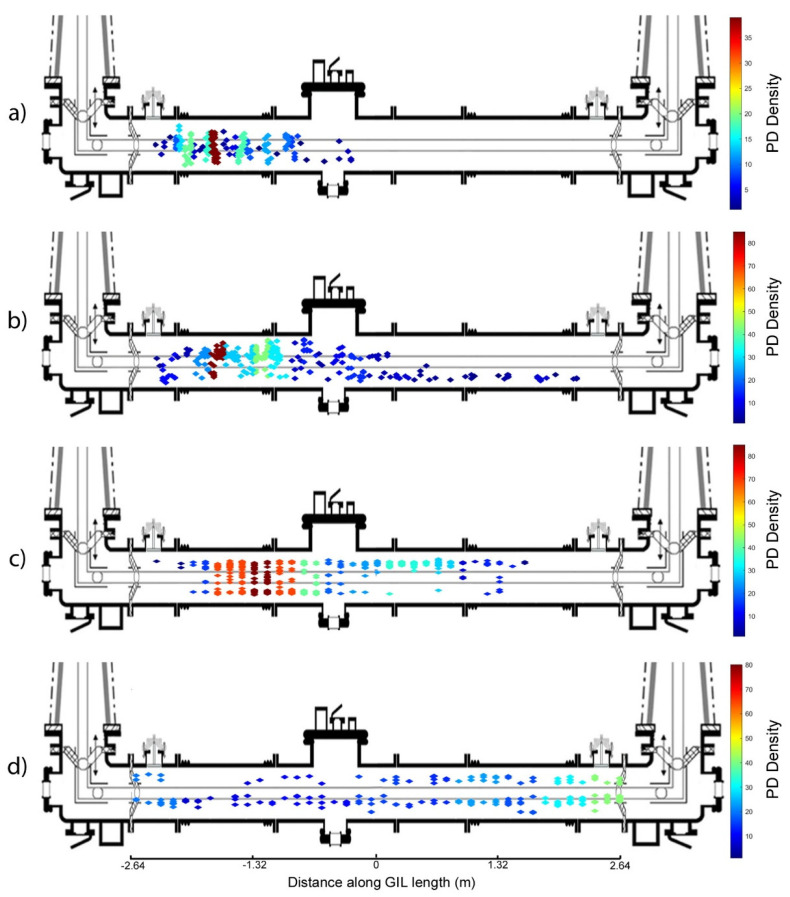
(**a**) PD UHF sensors used with CO_2_ at 3 bar with an applied voltage of 150 kV in a clean system; (**b**) PD UHF sensors used with CO_2_ at 3 bar with an applied voltage of 85 kV in a particle contaminated system; (**c**) PD HFCT sensors used with CO_2_ at 3 bar with an applied voltage of 85 kV in a particle contaminated system; (**d**) PD HFCT sensors used with CO_2_ at 3 bar with an applied voltage of 100 kV in a particle contaminated system.

The next section of this paper uses the above method to calculate the time difference of the arrival of each PD event at either the UHF sensors or the HFCTs. For each gas and pressure described below, many PD events were recorded and their location and grouping of PD signals has been plotted, the scatter plot on the left shows the highest peak voltage level obtained from the HFCT or UHF sensors and the time difference (proportional to location) of the PD event along the central section of the GIL. The colour of the points shown on the scatter plots (Figure 10, Figure 11, Figure 12, Figure 13 and Figure 14) show the amount or density of PD along the center of the GIL with red points indicating areas with the highest amount of PD activity. The histogram plot shown on the right side of Figure 10, Figure 11, Figure 12 and Figure 13 shows the amount of PD activity along the central section of the GIL, with the areas of highest PD activity shown with the highest number of PD counts recorded.

In Figure 10, the PD events that were recorded using the two UHF sensors at either end of the GIL demonstrator are shown when it is filled with technical air in a clean system (no particles have been introduced into the enclosures). Figure 10a,b show the PD activity of the system when a voltage of 210 kV is applied to the central conductor at 3 bar pressure. Figure 10c,d shows the PD events recorded when the gas insulated enclosures are filled with 5 bar technical air and a voltage of 200 kV is applied. Figure 10e,f shows the PD events recorded when a voltage of 231 kV is applied to the GIL demonstrator when filled with 5 bar technical air. In Figure 10, for technical air, there are very few PD events recorded even at the normal phase to earth voltage of the system of 231 kV. Examining Figure 10a,c for both 3 bar and 5 bar technical air at 210 kV and 200 kV, respectively, the results are shown for two different pressures; however, there was very little difference observed because very little PD was recorded for technical air overall for all tests carried out in the clean system. For all results recorded in Figure 10b,d,f, there seems to be a similarity with most PD events recorded around the −20 to −10 ns range which relates to the central left of the GIL demonstrator, so this appears to be an area of high electric field stress but there are very few PD events from which to draw this conclusion.

Figure 11 shows the PD activity recorded by the two internal UHF sensors placed at either end of the GIL demonstrator as shown in Figure 5, the GIL was filled with 3 bar pure CO_2_. The applied voltage was 150 kV on the clean system and the response of the UHF sensors is shown in Figure 11a,b. The GIL demonstrator was then tested using the same conditions, except the central enclosure was contaminated with particles and the voltage raised to 85 kV; the results of which, for the UHF sensors, are shown in Figure 11c,d. In Figure 11b,d, there is a strong clustering of PD events in the central left of the GIL demonstrator. In Figure 11a, it is interesting to note that for a clean system the grouping of PD events from the internal capacitive UHF plate sensors has a max voltage relating to the PD events which are very closely grouped, whereas in Figure 11c, for a particle contaminated system, there is a much more varied voltage response. This means that the particles are creating a very different response from the UHF sensor as they build charge moving across the CO_2_ insulation between the conductor and enclosure at different lengths along the GIL, which does not occur for the clean system. The moving particles also appear to have the impact that they further exacerbate the electric field stress on the left side of the GIL demonstrator shown in the clean system response; hence, the area of highest PD response is the same for both a clean and a particle contaminated system.

The next set of experimental data shown in Figure 12 shows the PD activity measurements using the HFCTs placed at either end of the GIL demonstrator. In Figure 12, the GIL demonstrator is filled with pure CO_2_ at 3 bar and the GIL demonstrator is contaminated with metal particles. The results show the difference in PD events as the voltage is increased. Figure 12a,b show the recorded PD events for 75 kV, Figure 12c,d show the recorded PD events for 85 kV, and Figure 12e,f show the recorded PD events for 100 kV. Figure 12a,c,e show that as the voltage on the central conductor is increased so does the PD amplitude from the HFCTs in quite distinct steps. For 75 kV on the central conductor, the majority of PD is around a 45 mV peak response from the HFCT; for 85 kV, the PD has a peak response of 70 mV from the HFCT; and for 100 kV, the PD peak amplitude is around 240 to 340 mV, which is directly proportional to the current the HFCT is measuring around the earth straps from the GIL outer enclosure for each PD event.

Figure 12b,d show that at 75 kV, the majority of PD events are located on the central left hand side of the GIL demonstrator, and for 85 kV, the majority of PD is located in a similar area on the left of the GIL with an added area on the right hand side which is not as active. When the voltage of conductor in the GIL demonstrator is raised to 100 kV, it appears that the contaminating metal particles are causing widespread PD along the entire central length of the conductor as shown in Figure 12f. It is possible that this indicates that the particles are much more mobile and either were bouncing on the outer enclosure along the entire length of the GIL or appear to have an aspect of freer movement in the gas along the length of the GIL. In Figure 12b,d, it is possible that the particles were only bouncing on the outer enclosure as described in [30], where a free particle may cause a discharge as charge is exchanged between the particle and the enclosure; following this, the particle falls and bounces on the enclosure and the sequence may repeat [30].

In Figure 13, the PD events of the UHF sensors are directly compared with the HFCT sensors by recording the same PD events simultaneously and then calculating time-of-flight from both sets of sensors. In Figure 13a,b, the recorded PD events for the UHF sensors are shown for the GIL demonstrator filled with pure CO_2_ at 3 bar when contaminated with metal particles at 85 kV applied voltage. In Figure 13c,d, the same conditions of pressure, gas and contamination are used whilst the same PD events for Figure 13a,b are measured and shown but using the HFCTs as PD sensors rather than the UHF sensors. Figure 13b,d show a comparison between the PD locations that are derived from the UHF sensors and the HFCT sensors—the results indicate that the main area of PD on the left of the GIL is fairly similar but that the HFCT sensors indicate a slight area of additional PD on the right of the GIL demonstrator which is not as apparent on the measurements taken using the UHF sensors. Figure 13a,c show that for 85 kV applied voltage in a particle contaminated system, both the UHF and HFCT sensors indicate two main data clusters in both cases. The first cluster, which has an approximately constant amplitude and occurs across the whole GIL length, can be assumed to be free particles bouncing along the bottom of the GIL. The second cluster, in the middle of the first half of the GIL, is the same area seen on an uncontaminated system which indicates a source of higher electric field stress than the rest of the system. This seems to indicate that this location analysis can separate different types of defect and give a better understanding of underlying sources of PD when multiple PD sources are present, as is often the case for a real system.

## 4. Discussion

The results shown in the previous section give an interesting overview of the diagnostic information that can be obtained from a high voltage gas insulated system in both clean and particle contaminated configurations with partial discharge in natural gases such as carbon dioxide and technical air. Figure 14 gives a summary of the previous important results for the purpose of discussion superimposed on top of the gas insulated demonstrator sections used for experimentation in carbon dioxide at 3 bar pressure.

Figure 14a,b show the difference in PD location using UHF sensors in CO_2_ when the gas insulated line is a clean system or contaminated with particles. It can be surmised from these results that the clean system in Figure 14a appears to have a high electric field stress region on the left side where the largest area of PD is indicated as a red region; however, the amount of PD events recorded for this case was much lower than for a contaminated system. In Figure 14b, the UHF sensors are used to record PD at a much lower voltage, 85 kV compared to 150 kV in Figure 14a, but the amount of PD recorded was much higher. Figure 14b seems to show that particle contamination appears to exacerbate the electric field concentration in the region identified in the clean system in Figure 14a and also shows that there were PD events further along the whole length of the central gas insulated line. The observed PD is consistent with the expected behaviour of free metallic particles in GIL free motion, where particles bounce on the outer enclosure of a section [31] when measurements are made using UHF sensors.

Figure 14b,c can be used to analyse the differences between the UHF and HFCT sensors; for the example shown, 85 kV is applied on the central conductor insulated by 3 bar CO_2_ and both UHF and HFCT sensors are used to measure the same events. The UHF results shown in Figure 14b show a clear high-density cluster of PD with an area just to the right of this also indicating a high PD concentration when contaminated with particles. The HFCT sensors in Figure 14c show a very similar result to the UHF sensors; however, the area is slightly shifted to the right and there is also a small amount of PD indicated on the right side of the GIL. The likely difference between the UHF and HFCT sensors is that the UHF sensors have a higher bandwidth therefore can detect a broader range of PD events; however, the HFCTs appear to be a good compromise considering they do not need to be permanently installed on the inside of the GIL as internal sensors, and they do not require the high level of associated measurement hardware i.e., high bandwidth oscilloscope. It is also possible that the UHF sensors are more accurate as they are internal and therefore should pick up less noise/interference from the surroundings. The UHF sensor also appears to be less sensitive to the free particle cluster than the other (unknown) defect which is constant in the clean system, and the HFCT is more sensitive to the free particle cluster than the unknown defect producing the small lower cluster. This observation could point to the benefits of using a combination of PD sensors that respond differently to PD events in order to gain more insight into the shape of the underlying PD pulse and hence the type of defect.

The last comparison can be made between Figure 14c,d, where HFCTs are used to measure PD in CO_2_ at 3 bar. In Figure 14c, the applied conductor voltage was 85 kV, and in Figure 14d, the applied voltage was 100 kV. In Figure 14c, the contaminating particles appear to enhance the areas of highest PD around a very similar location to the areas of high field stress as shown in the clean GIL test results shown in Figure 14a. In Figure 14d, the PD appears to spread uniformly across the whole length of the central GIL section in which the contaminating particles are placed. This seems to indicate that PD events from the free-moving particles are dominant in amplitude and that the particles are either bouncing on the enclosure along the whole section or some free movement occurs at 100 kV, whereas at 85 kV, the particles may not have attained sufficient energy to move from their location in the enclosure and so cause this effect on the PD sensors. At 85 kV, the pattern detected is not consistent with those of free conducting particles in gas insulated equipment where a particle becomes charged by induction under the influence of the power-frequency electric field and may discharge anywhere along the system length. This indicates that the particles measured are close to free mobility on the inner enclosure of the gas insulated line demonstrator at 100 kV, but would not present a deleterious threat to the insulation system at these voltages unless they were to become trapped in the spacer-chamber junction.

## 5. Conclusions

The results of the practical research conducted in this paper show that CO_2_ and technical air can be used to insulate a gas section normally insulated with SF_6_ at phase-to-earth voltages of 242 kV at a similar pressure to SF_6_; however, increased pressure is likely to reduce the amount and impact of PD and increase the longevity and lifetime of the equipment. The research also concluded that both HFCT and UHF sensors can be used to detect partial discharge with natural gases with internal UHF sensors showing the most accurate PD location results, but external HFCTs offer a good compromise between cost and location accuracy for a PD detection system. This potentially demonstrates their future application in gas insulated equipment, especially for large substations where accurate PD location within the GIL would require PD sensors to be installed in each GIL subsection. In this paper, it has been shown that the addition of a second UHF and HFCT allows for the added dimension of spatial location measurements. This added location measurement allows for significantly more information to be yielded than a single sensor, including the ability to locate multiple, simultaneous active defects by identifying clusters in the PD number and location plot. Initial indications from these measurements are that, due to the different ways in which direct and UHF measurements respond to the same PD event, the simultaneous measurement of both methods along with quantities along the physical location of the PD with the GIL could yield even further information about the physics of the discharge event, leading to more accurate diagnostics for future gas insulated systems. The PD results in this paper could also be used to benchmark the PD characteristics of future gas insulation mixtures that are currently being trialed to replace SF_6_, as many of these consist of high ratios of CO_2_. Future research will determine the PD system response of other natural atmospheric gases filled in conventional high voltage gas insulated equipment, including increased pressure ratings.

## Figures and Tables

**Table 1:** Gas insulated line demonstrator Rated and Nominal System Voltage Levels.

U_r_ Gas Insulated Line Demonstrator Rated Voltage	U_ph-ea_ Nominal System Phase to Earth Voltage	U_pd-test_ Test Voltage for PD Measurement U_ph-test_ (>1 min)
300 kV	173 kV	208 kV
362 kV	209 kV	251 kV
400 kV	231 kV	277 kV
420 kV	242 kV ^1^	291 kV

^1^ Maximum applied voltage achieved during practical testing was 242 kV due to bushing air corona.

**Table 2:** Gas insulated line demonstrator withstand voltages in clean conditions.

	3 bar	4 bar	5 bar
**Pure Carbon Dioxide (CO_2_)**	>242 kV	>242 kV	>242 kV
**Pure Nitrogen (N_2_)**	>192 kV	Breakdown (BD) at 211 kV and 241 kV	>242 kV
**Technical Air**	>242 kV	>242 kV	>242 kV
